# Supplementary material for: RNA degradation by the plant RNA exosome involves both phosphorolytic and hydrolytic activities
Source: Nat Commun. 2017 Dec 18;8:2162. doi: 10.1038/s41467-017-02066-2 (PMC5735172; doi:10.1038/s41467-017-02066-2)
Supplement: Supplementary file 1 — Supplementary Information [file 41467_2017_2066_MOESM1_ESM.pdf]

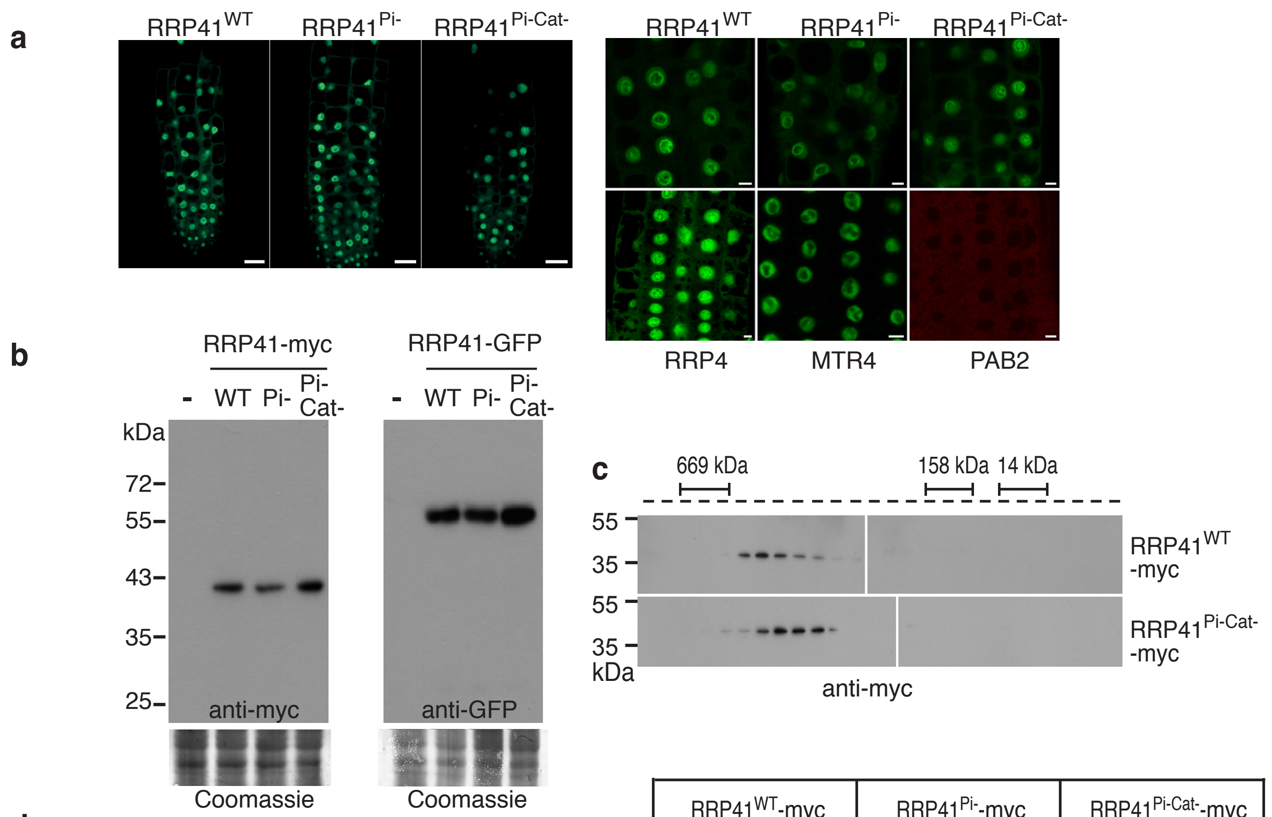

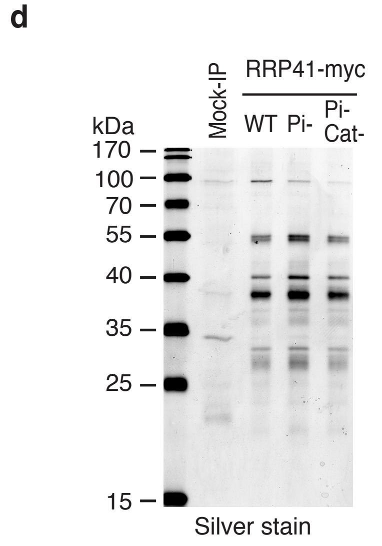

		RRP41^{WT}-myc		RRP41^{Pi-}-myc		RRP41$^{Pi-Cat-}$-myc	
Accession	Name	Mascot score	# Spectra	Mascot score	# Spectra	Mascot score	# Spectra
At1g03360	RRP4	1586	713	1793	750	1519	745
At1g60080	RRP43	2172	279	2444	310	2247	296
At2g25355	RRP40A	1804	183	1974	198	1769	208
At3g07750	RRP42	1677	363	1653	431	1705	369
At3g46210	RRP46	780	74	875	97	695	81
At3g12990	RRP45A	778	58	777	47	710	50
At3g60500	RRP45B	2394	273	2484	317	2264	299
At3g61620	RRP41*	1765	354	1444	265	1257	251
At4g27490	MTR3	1741	211	1753	235	1729	235
At5g38890	CSL4	1542	283	1672	311	1432	299

Supplementary Fig. 1. Characterization of wild type and mutated RRP41 proteins

(**a**) Intracellular localization of RRP41^{WT}, RRP41^{Pi-}, and RRP41$^{Pi-Cat-}$ fused to GFP in roots of stable Arabidopsis transformants. All fusion proteins are detected with similar fluorescence intensities and are localized in both the nuclear and cytoplasmic compartments. A close-up view on the right shows also GFP fused to the exosome subunit RRP4 as well as MTR4-GFP and PAB2-RFP as nucleolar and cytosolic markers, respectively. Scale bars are 20 and 5 μm for left and right panels, respectively.

(**b**) Western blot showing a similar level of expression between wild type and mutated RRP41 proteins in the myc or GFP transgenic lines.

(**c**) Both wild type and mutated RRP41 proteins are incorporated in high molecular weight complexes. Total protein extracts from RRP41^{WT} and RRP41$^{Pi-Cat-}$ plants were analyzed by gel filtration followed by SDS-PAGE and western blot with an anti-myc antibody. The elution profile of size markers for the gel filtration is indicated above the fractions, the size markers for the SDS-PAGE are indicated on the left. RRP41-myc is only detected in high molecular weight complexes but not as a monomer.

(**d**) Characterization of immunoprecipitated Exo9s having incorporated wild type or mutated myc-tagged RRP41 proteins. On the left, immunoprecipitated fractions were analyzed by SDS-PAGE followed by silver staining. A mock immunoprecipitation (IP) was performed using untransformed Col-0 plants. On the right, mass spectrometry analysis for Exo9 subunits shows that similar levels of Exo9 proteins are purified alongside wild type or mutated RRP41 prōteins fused to myc. The bait, RRP41, is marked with an asterisk. As previously observed[1], low amounts of the known co-factors HEN2, MTR4 and RRP44 co-purified under these experimental conditions and to a similar extent for wild type and mutated RRP41s. Full mass spectrometry data have been deposited to the ProteomeXchange Consortium via the PRIDE[2] partner repository with the dataset identifier PXD007984.

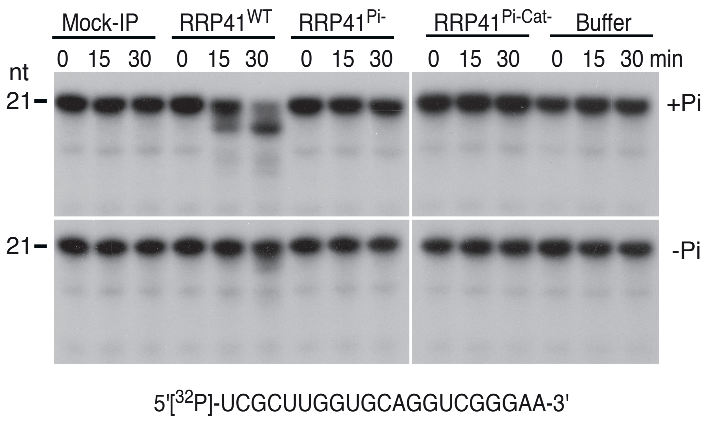

Supplementary Fig. 2.
Degradation of heteropolymeric RNA substrates by Exo9's phosphorolytic activity
Exo9s immunopurified from Col-0 (mock-IP), RRP41^{WT}, RRP41^{Pi-} and RRP41$^{Pi-Cat-}$ plants, were incubated with a 5'[^{32}P]-labeled 21 nt RNA substrate in the presence or absence of inorganic phosphate (Pi), followed by separation of products by PAGE and autoradiography. The sequence of the RNA substrate is given below the panel.

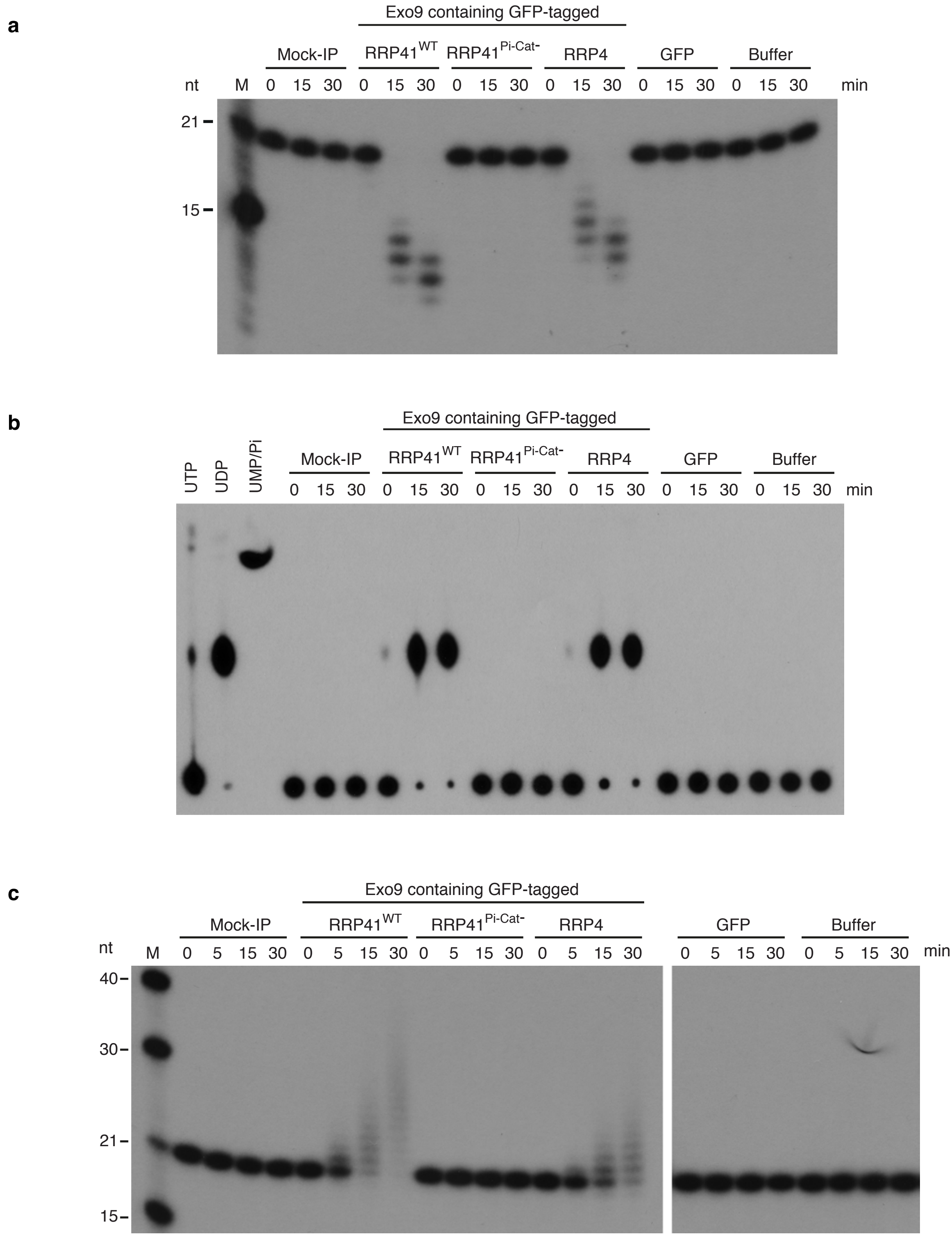

Supplementary Fig. 3. Activity of Exo9 complexes purified with either tagged RRP41 or RRP4 subunits
Exo9 complexes were purified from plant expressing either active or inactive versions of GFP-tagged RRP41, or a GFP-tagged version of the RNA binding protein RRP4, a subunit of the exosome cap. Plants expressing GFP were included as an additional control. (**a**) A 5' [^{32}P]-labeled oligo(U)21 RNA substrate was incubated in the presence of 3.5 mM Pi for the indicated times with a mock-IP fraction or immunopurified Exo9 complexes purified with the GFP-tagged versions of the subunits indicated on the top. GFP immunoprecipitation from plants expressing GFP alone were included as additional negative control. Reaction products were run on a denaturing acrylamide gel and analyzed by autoradiography.
(**b**) A 3' [^{32}P]-labeled oligo(U) RNA substrate was incubated with a mock-IP fraction or immunopurified Exo9 complexes as indicated on the top. Reaction products were analyzed by thin layer chromatography to separate nucleoside mono-, di- and tri-phosphate.
(**c**) *In vitro* RNA synthesis assays were performed as in **a** except that Pi was replaced by 1 mM UDP.

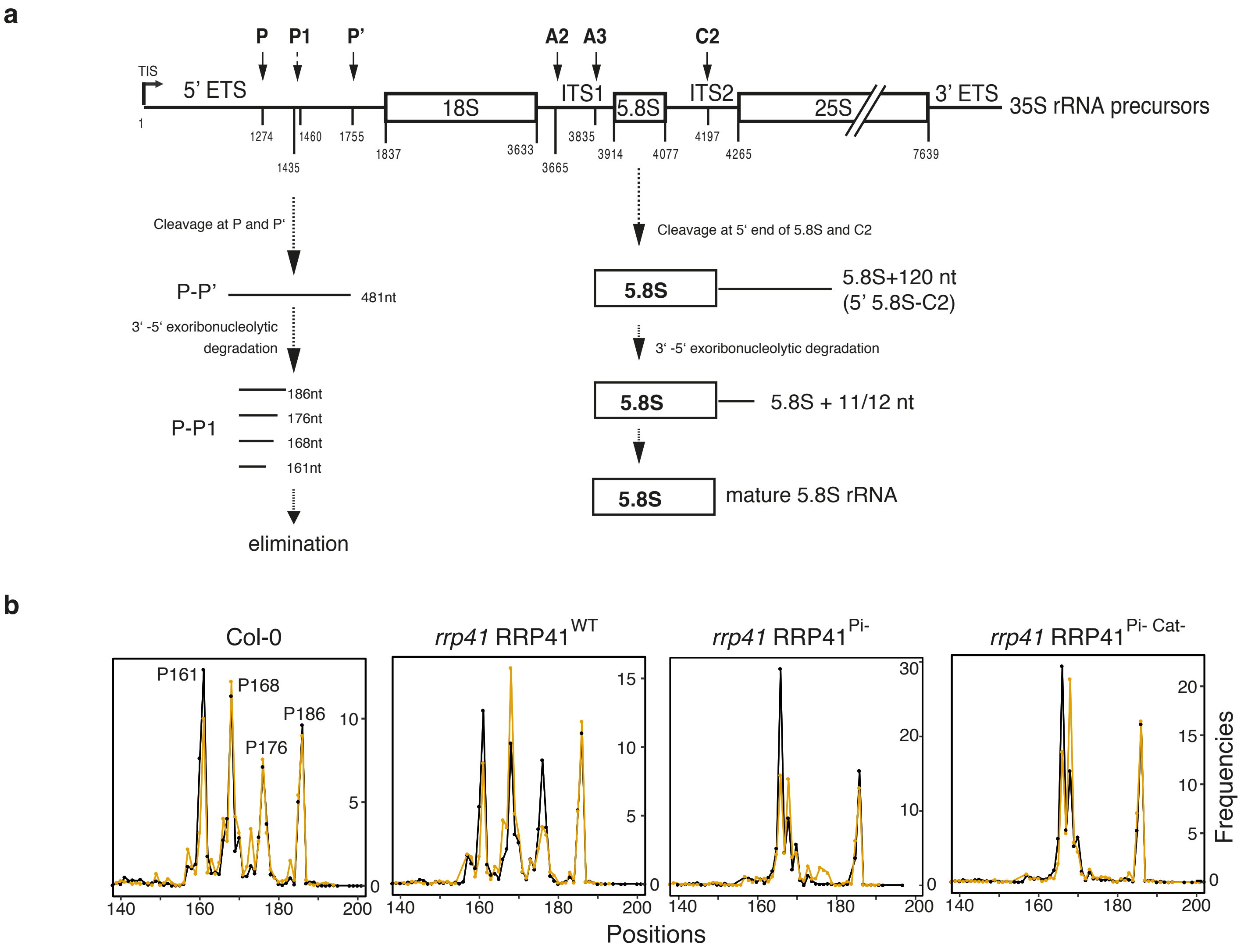

Supplementary Fig. 4. Impact of wild type and mutated RRP41 proteins on the degradation of P-P1 intermediates
(**a**) Diagram of the Arabidopsis 35S rRNA precursor (genbank accession X52322). 18S, 5.8S and 25S rRNAs (boxes) are separated by internal transcribed sequences (ITS) 1 and 2, and flanked by 5' and 3' external transcribed spacers (ETS). Numbers refer to the transcription start site. Vertical arrows above the diagram indicate the known processing sites. Main steps leading to the elimination of the 5' ETS and the production of mature 5.8S rRNA are depicted below.
(**b**) High density mapping by 3'RACE-seq of 3' extremities for P-P1 degradation intermediates. Density profiles are shown for positions +140 to +200, +1 corresponding to the first nucleotide of the P-P' fragment. The four main P-P1 intermediates are noted P161, P168, P176 and P186 in the left panel. Two replicates are shown in black and orange. Identical P-P1 intermediates are detected in Col-0 and RRP41^{WT}, showing a full complementation of the *rrp41* mutation by the RRP41^{WT} transgene. In addition, highly similar profiles are observed between *rrp41* RRP41^{Pi-} and *rrp41* RRP41$^{Pi-Cat-}$, indicating that both mutated RRP41 proteins are inactive.

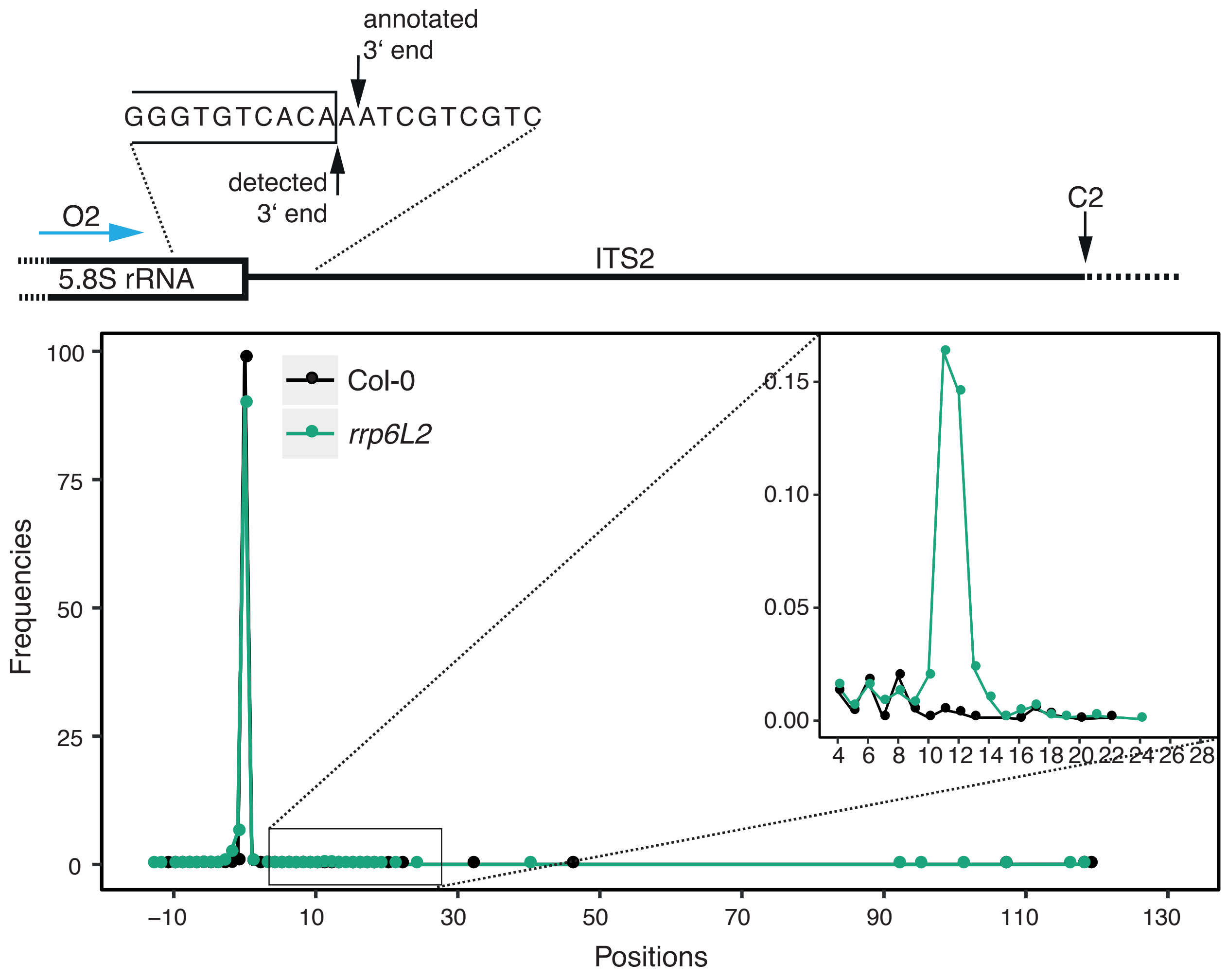

Supplementary Fig 5. Mapping the 3' extremities of mature 5.8S rRNA
High density mapping of 5.8S rRNA extremities in Col-0 and *rrp6L2*. Identical extremities are detected in Col-0 and *rrp6L2,* while the 5.8S + 11/12 nt precursors are detected only in *rrp6L2*

.

Supplementary Fig. 6.
Rosette phenotypes of Exo9 activity mutants
Rosettes of plants expressing endogenous RRP41, RRP41^{WT} or RRP41$^{Pi\text{-}Cat\ -}$ in Col-0, *rrp6L2*, *RRP44KD rrp6L2* or *mtr4* genetic backgrounds. (**a**) plants were sown directly in individual pots and grown for six weeks in 12h light / 12h darkness. (**b**,**c**,**d**) Plants were germinated and grown for 2 weeks in 12h light / 12h darkness, outbedded to individual pots and grown for further 4 weeks in 16h light / 8h darkness.

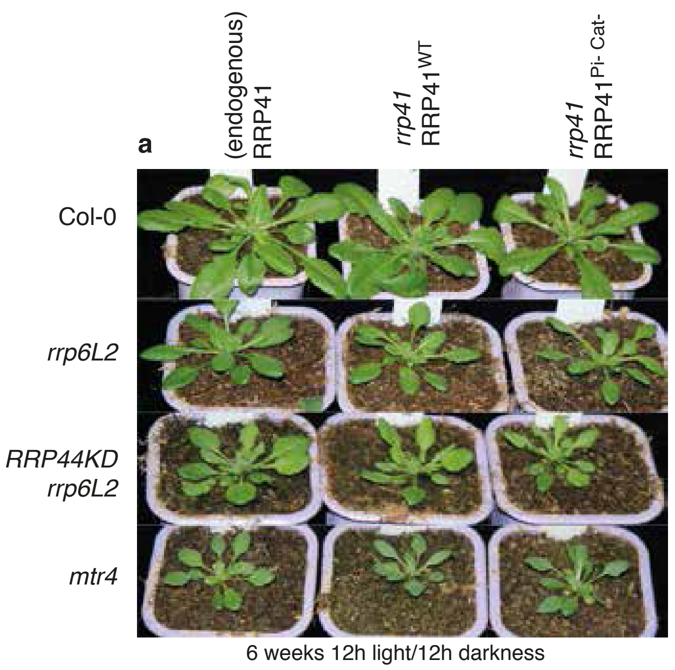

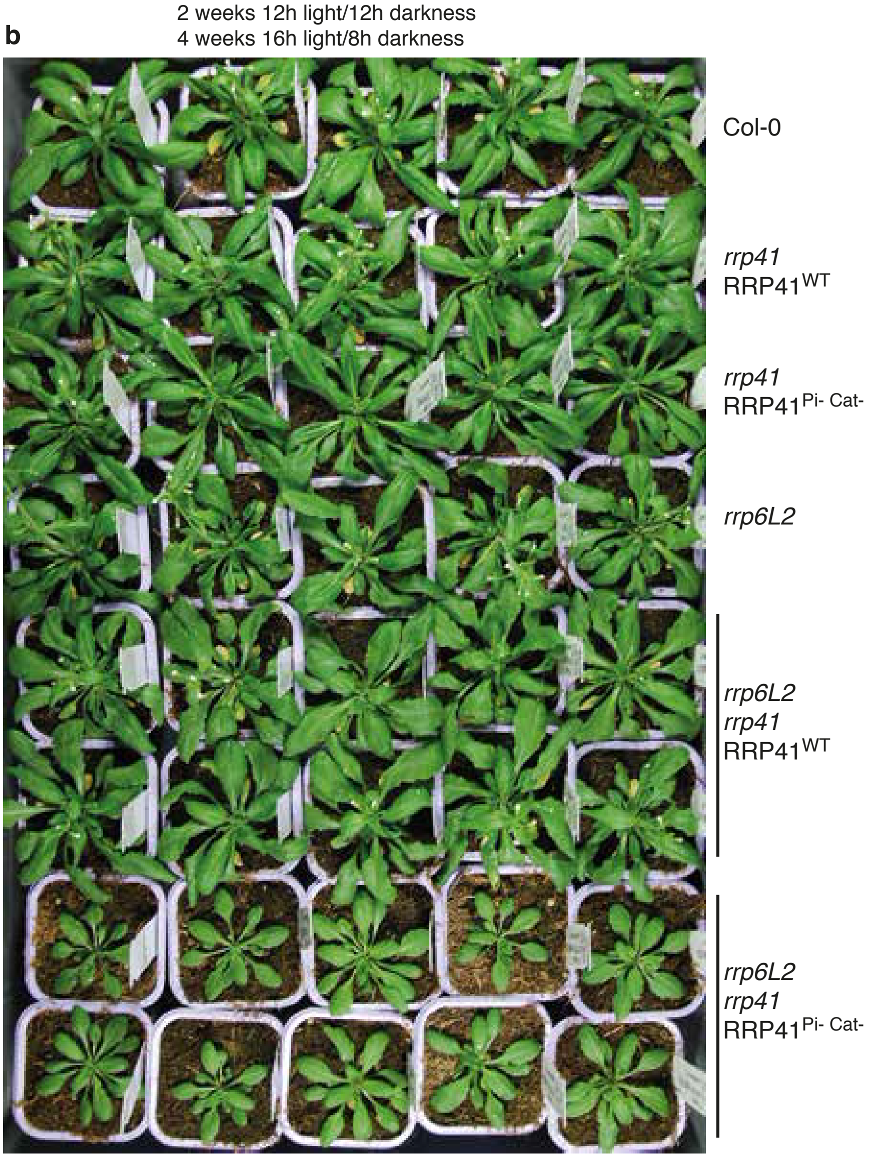

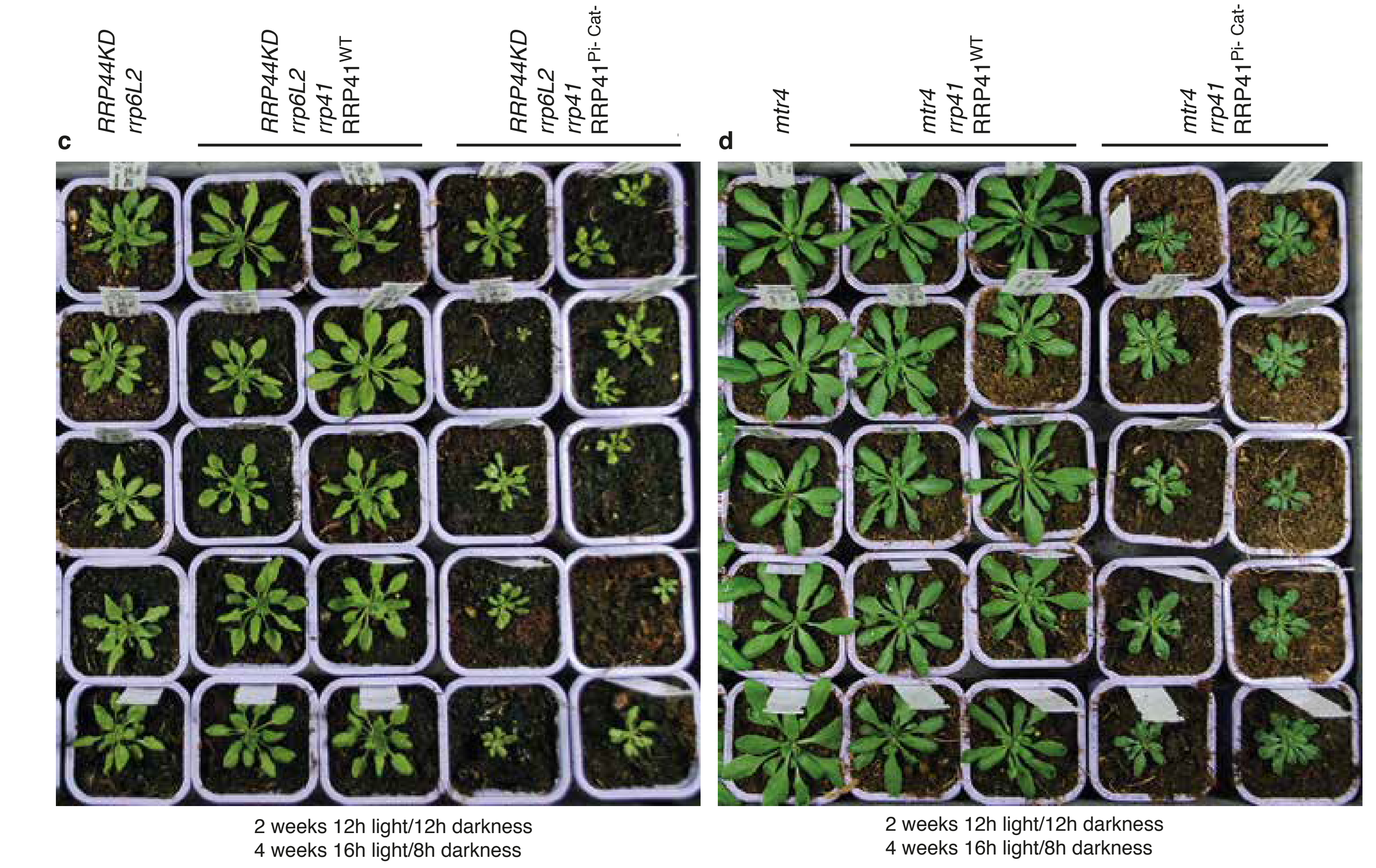

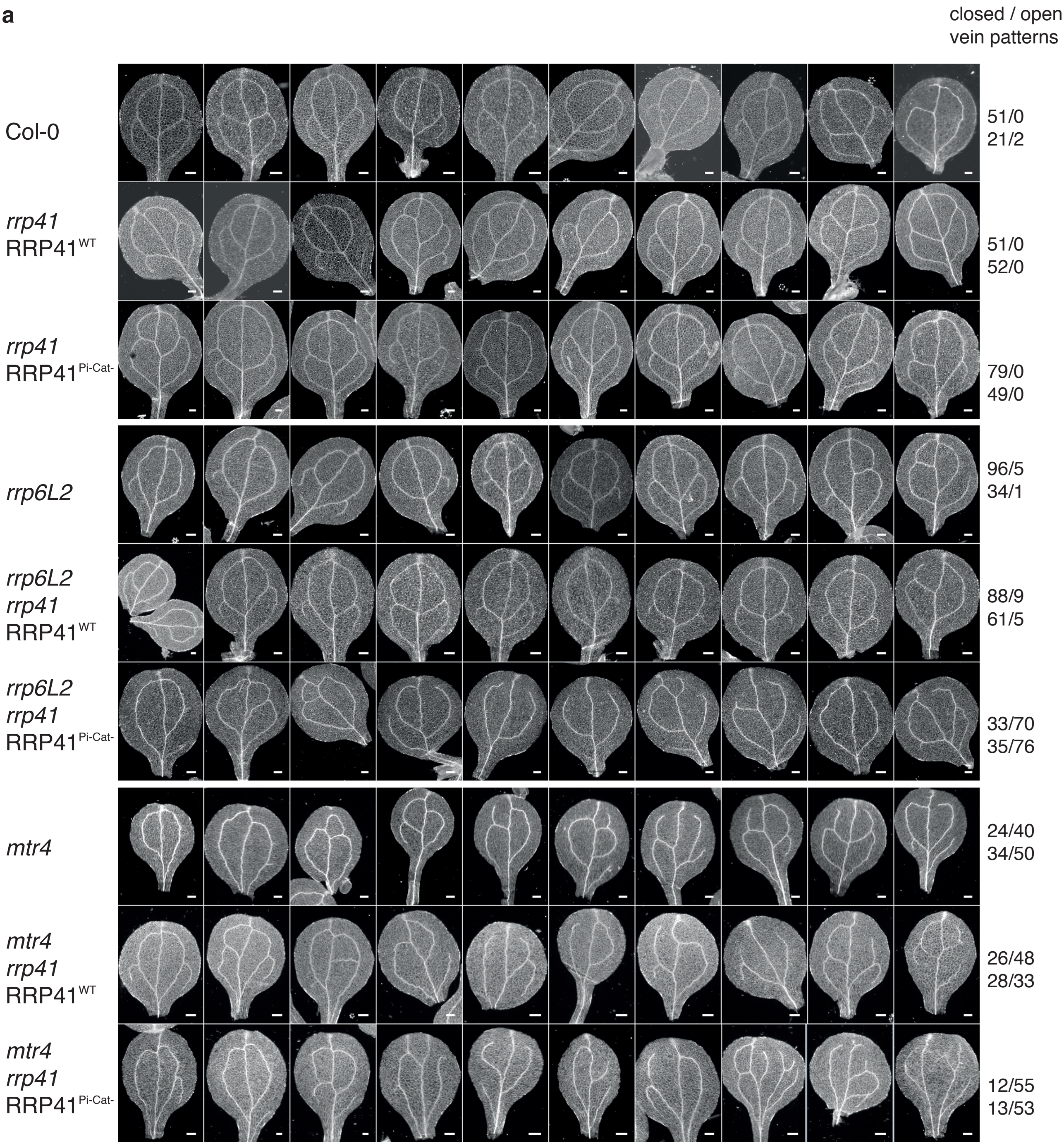

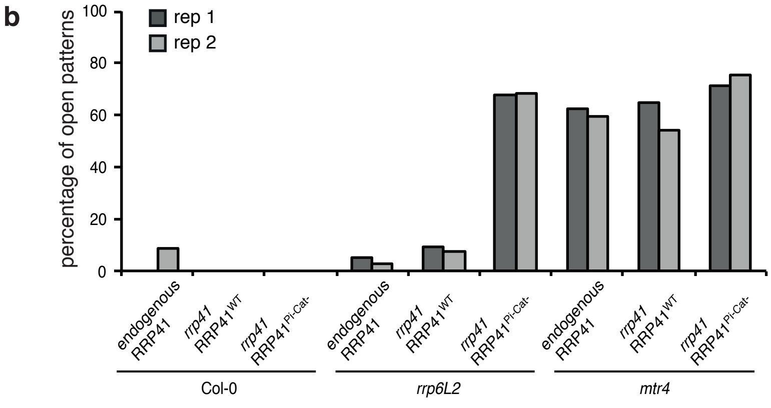

Supplementary Fig. 7. Plants lacking the catalytic activities of Exo9 and RRP6L2 have defective cotyledon vein patterns indicating impaired ribosome function or biogenesis. (**a**) Vein patterns of cotyledons taken from 7-10 day old seedlings were examined by darkfield microscopy. Ten representative examples are shown for each genotype. Scalebars are 200 μm. (**b**) Histogram showing the proportion of defective cotyledon vein patterns in indicated genotypes for two biological replicates. The total number of cotyledons with the earliest veins forming closed/open patterns are given on the right of panel **a** for two biological replicates.

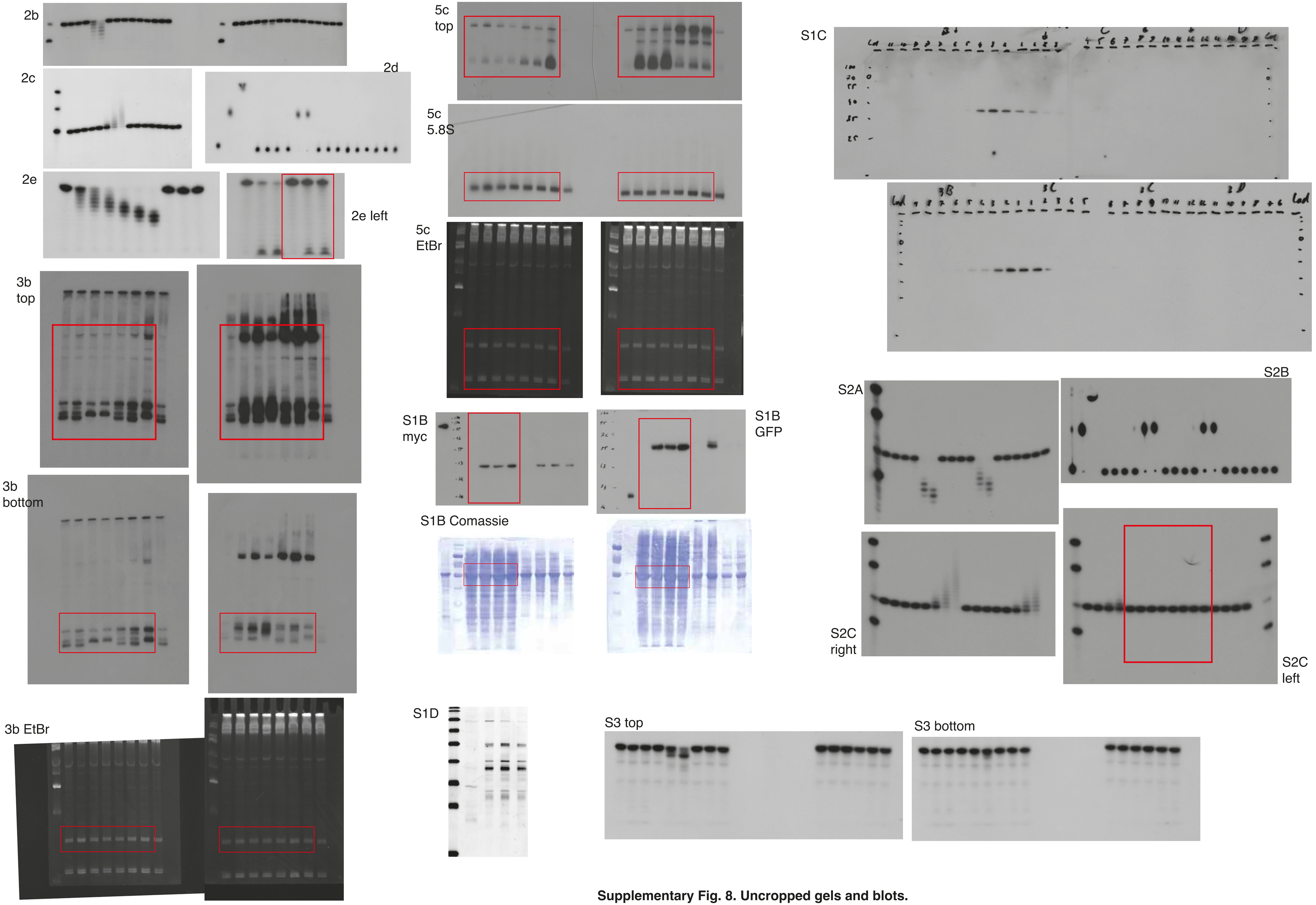

Supplementary Fig. 8. Uncropped gels and blots.

Supplementary Table 1. Number of reads mapping the 5' ETS (External Transcribed Spacer) of the 18S-5.8S-25S rRNA primary transcript across genotypes and replicates (Rep).

TruSeq Index	Genotype	Rep	5' ETS matching reads	Dedupli-cation	Read 2 with delimiter	Final number of reads
Index 9	Col-0	1	21026	18508	14082	11729
Index13	*rrp41* RRP41^{WT}	1	15156	13603	9194	7135
Index37	*rrp41* RRP41^{Pi-}	1	4762	4206	2095	1621
Index14	*rrp41* RRP41$^{Pi-Cat-}$	1	17270	15422	10445	8469
Index15	*rrp6L2*	1	15280	14028	9083	7371
Index16	*rrp6L2 rrp41* RRP41^{WT}	1	40620	35793	28958	23152
Index17	*rrp6L2 rrp41* RRP41$^{Pi-Cat-}$	1	15510	14171	8032	6046
Index32	*RRP44KD rrp6L2*	1	36238	34160	27985	22465
Index33	*RRP44KD rrp6L2 rrp41* RRP41^{WT}	1	22469	19803	14394	11364
Index34	*RRP44KD rrp6L2 rrp41* RRP41$^{Pi-Cat-}$	1	35918	30297	23525	18434
Index5	*mtr4*	1	87111	75701	12178	8234
Index7	*mtr4 rrp41* RRP41^{WT}	1	34533	30174	20069	16513
Index11	*mtr4 rrp41* RRP41$^{Pi-Cat-}$	1	18545	15973	9828	7813
Index8	Col-0	2	421313	213200	8403	1634
Index19	*rrp41* RRP41^{WT}	2	61594	31137	5281	3174
Index38	*rrp41* RRP41^{Pi-}	2	252463	177435	7784	1639
Index20	*rrp41* RRP41$^{Pi-Cat-}$	2	7277	6734	4828	3868
Index21	*rrp6L2*	2	44143	32052	11574	9029
Index22	*rrp6L2 rrp41* RRP41^{WT}	2	38633	29044	10209	7818
Index23	*rrp6L2 rrp41* RRP41$^{Pi-Cat-}$	2	62454	46680	10839	7225
Index27	*RRP44KD rrp6L2*	2	54578	44742	14174	10843
Index28	*RRP44KD rrp6L2 rrp41* RRP41^{WT}	2	10845	9453	5187	4208
Index29	*RRP44KD rrp6L2 rrp41* RRP41$^{Pi-Cat-}$	2	14116	10553	3916	2996
Index12	*mtr4*	2	25780	22305	11023	8745
Index35	*mtr4 rrp41* RRP41^{WT}	2	21418	17109	8076	6171
Index36	*mtr4 rrp41* RRP41$^{Pi-Cat-}$	2	35977	21881	8529	6658

Supplementary Table 2. Number of reads mapping the 5.8S rRNA sequence across genotypes and replicates (Rep).

TruSeq Index	Genotype	Rep	5.8S rRNA matching reads	Dedupli-cation	Read 2 with delimiter	Final number of reads
Index9	Col-0 (5.8S rRNA 3' mapping)	1	176785	176138	159281	146579
Index15	*rrp6L2* (5.8S rRNA 3' mapping)	1	176409	175918	154767	141562
Index 9	Col-0	1	80692	75140	53506	24560
Index13	*rrp41* RRP41^{WT}	1	66423	62844	40952	17166
Index14	*rrp41* RRP41$^{Pi\text{-}Cat\text{-}}$	1	118308	113266	75259	31902
Index15	*rrp6L2*	1	72050	70816	53621	38794
Index16	*rrp6L2 rrp41* RRP41^{WT}	1	380808	348651	253387	179214
Index17	*rrp6L2 rrp41* RRP41$^{Pi\text{-}Cat\text{-}}$	1	75199	74430	57139	38709
Index32	*RRP44KD rrp6L2*	1	166757	162980	131432	95155
Index33	*RRP44KD rrp6L2 rrp41* RRP41^{WT}	1	130136	126438	99739	69516
Index34	*RRP44KD rrp6L2 rrp41* RRP41$^{Pi\text{-}Cat\text{-}}$	1	160816	156686	123714	82297
Index37	*mtr4*	1	115970	115086	96933	54386
Index7	*mtr4 rrp41* RRP41^{WT}	1	151858	149677	128842	80336
Index11	*mtr4 rrp41* RRP41$^{Pi\text{-}Cat\text{-}}$	1	105689	103693	86558	48229
Index30	Col-0	2	31790	22526	16808	7924
Index31	*rrp41* RRP41^{WT}	2	59673	33205	24266	10422
Index32	*rrp41* RRP41$^{Pi\text{-}Cat\text{-}}$	2	78940	74332	51613	22961
Index21	*rrp6L2*	2	66444	63378	54345	44058
Index22	*rrp6L2 rrp41* RRP41^{WT}	2	65002	61890	51343	38004
Index23	*rrp6L2 rrp41* RRP41$^{Pi\text{-}Cat\text{-}}$	2	63068	61972	51851	37972
Index27	*RRP44KD rrp6L2*	2	89498	88677	75164	54740
Index28	*RRP44KD rrp6L2 rrp41* RRP41^{WT}	2	53018	52638	44373	32939
Index29	*RRP44KD rrp6L2 rrp41* RRP41$^{Pi\text{-}Cat\text{-}}$	2	80298	79326	65735	45042
Index12	*mtr4*	2	143794	142078	118315	76624
Index35	*mtr4 rrp41* RRP41^{WT}	2	102161	101039	84777	53908
Index36	*mtr4 rrp41* RRP41$^{Pi\text{-}Cat\text{-}}$	2	133971	132109	110699	71159

Supplementary Table 3. Custom DNA oligonucleotides used in this study.

Name	Sequence 5'-3'	Application
3'-Adap	/5rApp/-CTGACNNNNNNNNNNNNNNNTGGAATTCTCGGGTGCCAAGGC-/3ddC/	3' RACE-seq library preparation
3'-RT	GCCTTGGCACCCGAGAA	3' RACE-seq library preparation, cDNA synthesis
O1	AATGATACGGCGACCACCGAGATCTACACGTTCAGAGTTCTACAGTCCGACGATCATCTCGCGCTTGTACGGCTTTG	3' RACE-seq library preparation, forward PCR primer for P-P1 fragments
O2	AATGATACGGCGACCACCGAGATCTACACGTTCAGAGTTCTACAGTCCGACGATCTTCTGGCCGAGGGCACGTCTG	3' RACE-seq library preparation, forward PCR primer for mature 5.8S rRNA
O3	AATGATACGGCGACCACCGAGATCTACACGTTCAGAGTTCTACAGTCCGACGATCTCTGCCTGGGTGTCACAAATC	3' RACE-seq library preparation, forward PCR primer for 5.8S rRNA precursors
O4	ACGCCGAACGTTCAATTAACTC	Northern blot hybridization probe for P-P1 fragments
O5	AGGATGGTGAGGGACGACGATTT	Northern blot hybridization probe for 5.8S precursors
O6	GTATCGCATTTCGCTACG	Northern blot hybridization probe for the mature 5.8S

Supplementary References

1. Lange, H. *et al.* The RNA helicases AtMTR4 and HEN2 target specific subsets of nuclear transcripts for degradation by the nuclear exosome in Arabidopsis thaliana. *PLoS Genet.* **10,** e1004564 (2014).
2. Vizcaíno, J. A. *et al.* 2016 update of the PRIDE database and its related tools. *Nucleic Acids Res.* **44,** D447-456 (2016).
