# Supplementary material for: RNA degradation by the plant RNA exosome involves both phosphorolytic and hydrolytic activities
Source: Nat Commun. 2017 Dec 18;8:2162. doi: 10.1038/s41467-017-02066-2 (PMC5735172; doi:10.1038/s41467-017-02066-2)
Supplement: Supplementary file 3 — Description of Additional Supplementary Files [file 41467_2017_2066_MOESM3_ESM.pdf]

Description of Supplementary Files

File Name: Supplementary Data 1
Description: RRP41 protein sequences used for diversity analysis shown in Fig. 7.

File Name: Supplementary Data 2
Description: RRP41 protein sequences used for diversity analysis shown in Fig. 8.

File Name: Supplementary Data 3
Description: Scripts for 3’RACE-seq analysis.
